# Prognosis when using extracorporeal membrane oxygenation (ECMO) for critically ill COVID-19 patients in China: a retrospective case series

**DOI:** 10.1186/s13054-020-2840-8

**Published:** 2020-04-15

**Authors:** Yingchun Zeng, Zhongxiang Cai, Yunyan Xianyu, Bing Xiang Yang, Ting Song, Qiaoyuan Yan

**Affiliations:** 1 The Third Affiliated Hospital of Guangzhou Medical University, Guangzhou, China; 2 Renmin Hospital of Wuhan University, Wuhan, Hubei Province China; 3 School of Health Sciences, Wuhan University, Wuhan, Hubei Province China; 4 Union Hospital, Tongji Medical College, Huazhong University of Science and Technology, Wuhan, Hubei Province China

The World Health Organization (WHO) has characterized the disease, coronavirus disease 2019 (COVID-19), as a pandemic on March 11, 2020 (www.who.int). As of March 11, the WHO had recorded a total of 118,326 confirmed COVID-19 cases, with 4292 death cases (www.who.int). While the cumulative mortality of COVID-19 is 3.63%, COVID-19 has resulted in more death cases than SARS and MERS combined [1]. Within China, a total of 80,955 cases are confirmed, with 4257 severe cases in mainland China (www.nhc.gov.cn). In severe cases of COVID-19, patients experience rapid disease progression and can quickly progress to acute respiratory distress syndrome (ARDS) [2].

Based on this, when COVD-19 patients develop ARDS and mechanical ventilation cannot be improved, extracorporeal membrane oxygenation (ECMO) can be used [3]. As mortality rates among critically ill COVID-19 patients can be as high as 61.5% [4], ECMO may play a role in reducing mortality rates [5]. The indications of using ECMO are “For patients with severe ARDS, it is recommended to perform lung expansion. In the case of adequate human resources, prone positioning should be recommended for at least 12 hours per day for protective ventilation. If severe respiratory failure persisted, then ECMO should be started as soon as possible.” [6]

Worldwide data on prognosis when using ECMO to treat critically ill patients with COVID-19 infection are not available, and whether ECMO plays a role in reducing patient mortality rates is currently unknown. This research letter provides the first evidence of prognosis in treating critically ill COVID-19 patients with ECMO in China. These preliminary data were collected from two medical centers of Wuhan, China (Table 1). These data could be of considerable value in judging whether ECMO should be recommended as a salvage therapy for critically ill COVID-19 patients.

**Table 1:** Clinical characteristics and ECMO outcomes for critically ill COVID-19 patients (*N* = 12)

Variables	Mean (SD)	Range	*n*	%
**Age** (years)	50.9 (13.5)	35–76		
**Gender** (male)			11	91.7
**Comorbidities**				
Hypertension			1	8.3
Heart disease			1	8.3
Diabetes			1	8.3
Hyperthyroidism			1	8.3
**Key reason for ICU admittance**				
Dyspnea			11	91.7
Fever			10	83.3
Coma			1	8.3
**Treatment type**				
ECMO			12	100
Mechanical ventilation			12	100
Antibiotic treatment			12	100
Antiviral therapy			12	100
Glucocorticoid therapy			10	83.3
Supportive therapy based on symptoms			12	100
**Duration of ECMO use** (days)	11.3 (7.8)	3–28		
**ECMO prognosis**				
Improving without ECMO			3	25.0
Still alive with ECMO but two with coma			4	33.3
Dying			5	41.7

*Abbreviations*: *ECMO* extracorporeal membrane oxygenation, *ICU* intensive care unit

To date, the role of ECMO in the management of COVID-19 is unpromising. Nearly half of the patients treated with ECMO died from septic shock and multiple organ failure. The observed late complications included bleeding and infection. While the World Health Organization (WHO) interim guidelines and China’s national interim guidelines for the diagnosis and treatment of COVID-19 infection (sixth version) have made general recommendations for the use of ECMO for ARDS and critical COVID-19 infection [3, 5], the preliminary evidence available in mainland China does not support this general recommendation.

Certainly, understanding the risk-to-benefit ratio of performing ECMO on critically ill COVID-19 patients is dynamic as the course of this novel disease unfolds. The Chinese government covers all costs to treat patients with the COVID-19 infection, so the cost analysis of ECMO is to date unavailable in mainland China. However, an average ECMO procedure in the USA costs $73,122 USD, indicating that ECMO is a highly resource-demanding procedure [7]. Therefore, a further, larger sample size study and a comprehensive analysis of the medical value of using ECMO on COVID-19 patients are urgently required. Based on the two cohort case series in this study, nearly half of the critically ill COVID-19 patients with ECMO were dying from septic shock and multiple organ failure. As anticipated by MacLaren et al. [3], COVID-19 is a pandemic; all healthcare resources are stretched so that ECMO is not a therapy to be rushed to the frontline. Therefore, interim treatment guidelines [5, 6] of recommending ECMO for critically ill COVID-19 patients should be taken cautiously.

## Data Availability

The datasets used in the present study are available from the first author and corresponding authors on reasonable request.

## Abbreviations

ARDS: Acute respiratory distress syndrome
COVID-19: Coronavirus disease detected in 2019
ECMO: Extracorporeal membrane oxygenation
